# Bio-Economic Comparison of Pure Holstein, Simmental × Holstein, and Montbéliarde × Holstein Crossbred Dairy Cows

**DOI:** 10.3390/ani16040644

**Published:** 2026-02-17

**Authors:** Soheila Noormohammadi, Seyed Abbas Rafat, Sadegh Alijani, Karim Hasanpur, Hadi Esfandyari

**Affiliations:** 1 Department of Animal Science, Faculty of Agriculture, University of Tabriz, Tabriz 51666, Iran; 2 Independent Researcher, Chamerstrasse 56, 6300 Zug, Zurich, Switzerland; hadi.esfandyari@qualitasag.ch

**Keywords:** economic comparison, crossbreeding, milk production, profitability

## Abstract

The economic performance of Holstein, Montbéliarde × Holstein, and Simmental × Holstein cows was evaluated using a bio-economic model. The results showed differences in income and costs between crossbred and pure Holstein groups. The Montbéliarde–Holstein crossbreds had the highest milk income ($2223) and profitability ($1002) compared to the Simmental crossbreds and pure Holstein. In contrast, pure Holstein made the most money from selling extra heifers, while both crossbred groups, especially Simmental × Holstein, made more money from selling male calves. Crossbred systems had much lower annual maintenance costs. Simmental × Holstein had variable costs that were 22% lower than those of pure Holstein. Trait-based economic evaluations demonstrated that Montbéliarde crossbreds exhibited the highest economic value for milk production, while Simmental crosses were more susceptible to prolonged calving intervals. Both crossbred groups had higher birth weights and growth rates than Holstein cows. Overall, these results provide strong evidence that crossbreeding Holstein with Montbéliarde maximizes overall profitability, while Simmental × Holstein also offers notable economic benefits through reduced maintenance costs and improved growth performance.

## Simple Summary

Increasing productivity and profitability in dairy herds is a key goal for farmers. Crossbreeding Holstein cows with other breeds is a common strategy to achieve this. Three dairy cow groups of pure Holstein, Montbéliarde × Holstein, and Simmental × Holstein were evaluated in this study. Using a bio-economic simulation model, we estimated income, costs, and profitability for each group. The crossbreds of Montbéliarde × Holstein had the highest milk income and overall profit. Pure Holstein cows earned more from selling surplus female calves, while crossbred cows, particularly Simmental × Holstein, earned more from selling male calves and required lower maintenance costs. Crossbred cows also produced calves that grew faster and had higher birth weights. These findings suggest that strategic crossbreeding can improve both profitability and animal health, offering practical benefits for dairy cattle farmers.

## 1. Introduction

The economic evaluation of diverse dairy cattle genotypes is one of the most important challenges in genetic improvement programs [1]. For over half a century, genetic selection programs in dairy cattle have prioritized enhancements in milk yield and solids content, with the Holstein breed exhibiting extraordinary gains in production and thereby establishing itself as the preeminent global dairy genotype [2]. Nevertheless, this singular emphasis on production traits has engendered deleterious repercussions, particularly pronounced deficits in functional attributes that compromise herd health, longevity, and economic viability. While Holsteins are universally acclaimed for their exceptional milk output, the rigorous selection for yield has inadvertently precipitated genetic antagonisms, manifesting as diminished fertility and health resilience [3,4,5]. Purebred Holsteins routinely display suboptimal fertility profiles, evinced by protracted calving intervals, elevated days open, and augmented services per conception. Compounding these reproductive constraints, Holsteins exhibit heightened vulnerability to an array of health disorders encompassing mastitis, lameness, and metabolic aberrations that escalate veterinary expenditures, inflate culling rates, and erode lifetime productivity, notwithstanding their superior per-lactation yields [6,7,8]. Some researchers showed that pure Holsteins incur a markedly higher mastitis incidence relative to crossbreds. Consequently, higher heifer replacement rates are required, which can ultimately reduce overall herd profitability [9,10,11]. Over the preceding decade, genetic improvement paradigms in the United States and beyond have pivoted from production-centric objectives toward functional traits that underpin the economic efficacy of dairy systems [12,13]. Breeding goals in heterogeneous production contexts, e.g., South Africa, Brazil, and the Czech Republic, are finding the economic weights for fertility and longevity traits. In mitigation of these challenges, crossbreeding paradigms have surfaced as pragmatic interventions, harnessing heterosis and breed complementarity to ameliorate functional traits with minimal encumbrance to milk production [14,15].

Crossbred genotypes, notably those derived from Simmental × Holstein and Montbéliarde × Holstein mating, evince substantial ameliorations in cardinal functional metrics: enhanced fertility via abbreviated days open and superior conception rates; fortified udder integrity, with reductions in mastitis incidence approaching 15%; and augmented survival and longevity, culminating in protracted productive lifespans and incremented lactations [3,16]. These functional gains are the marginal concessions in per-cow milk yield estimations [17,18]. For instance, Hazel et al. (2021) documented that two- and three-breed crossbreds surpass pure Holsteins in herd longevity, mortality mitigation, and lifetime profitability, yielding annualized per-cow profit increments of $173 and $125, respectively [19]. Danish investigations have quantified heterosis economic value at 21% and 30% for two- and three-breed systems [2], while California field trials affirmed 4–5% higher daily net returns for Montbéliarde × Holstein and Scandinavian Red × Holstein crossbreds vis-à-vis pure Holsteins [20]. Recent inquiries corroborate that crossbreds consistently outperform pure Holsteins in net daily profitability, irrespective of parity [21]. However, optimal breeding regimens defy universal prescription, as they vary depending on farm-specific conditions, management protocols, and socioeconomic factors [22]. Bio-economic modeling frameworks thus emerge as indispensable for rigorous appraisal, amalgamating intricate biological parameters with pecuniary variables to extrapolate trait-specific economic values and forge equilibrated selection indices conducive to enduring sustainability [7].

The present study endeavors a critical, comparative economic dissection of pure Holstein, Simmental × Holstein, and Montbéliarde × Holstein crossbred dairy genotypes. This study elucidates the relative merits and trade-offs of these genetic constellations, furnishing data-informed perspicacity to calibrate breeding stratagems in modern dairy enterprises, with paramount emphasis on equilibrating productivity and functional robustness.

## 2. Materials and Methods

In this study, data were collected from the Moghan Agro-Industry and Animal Husbandry Company, Parsabad Moghan City, Ardabil Province, Iran, between 2018 and 2024. The company comprises five Holstein dairy stations, each with a capacity of 2200 lactating cows; two heifer-rearing stations with capacities of 3000 and 2000 head; a purebred bull-rearing station with a capacity of 3000 head; and a Simmental–Montbéliarde station with a capacity of 1200 head. The dairy herd includes approximately 6300 lactating cows producing over 190 tons of milk per day. The herd consists of Holstein, Jersey, Simmental × Holstein, and Montbéliarde × Holstein breeds. The farm produces 160,000 tons of corn silage and 24,000 tons of alfalfa annually. On average, 200 kg of nitrogen fertilizer per hectare is applied to corn forage fields.

In this study, a total of 600 cows, including Holstein (*n* = 198), Montbéliarde × Holstein (*n* = 210), and Simmental × Holstein (*n* = 192), were chosen for analysis. The animals included in the study were chosen using comprehensive herd records from the farm’s information system. We considered the accuracy and consistency of data regarding production, reproduction, health, and management. Cows were maintained under standardized industrial conditions, which included open housing with automatic waterers and sand-bedded resting areas. The barns were cleaned three times per day using scrapers. Milking was carried out three times daily in herringbone milking parlors. Feed was provided as a total mixed ration eight times per day, with the amount and composition adjusted according to the stage of production and body condition score. Drinking water was available ad libitum. Environmental variables such as temperature and humidity were continuously monitored, and misting systems were used during the summer to maintain thermal comfort. Health, reproductive, and production management followed unified company protocols, including regular veterinary examinations, vaccination programs, disease prevention, and monitoring of reproductive performance. The herd employs a terminal two-breed crossbreeding system. Pure Holstein females are inseminated with Montbéliarde or Simmental semen to deliver F1 crossbred offspring. A sufficient pure Holstein female base is maintained to produce all required F1 replacement heifers internally; no external female calves or heifers are purchased. Calves born to F1 cows (both male and surplus females) are sold. Full rearing costs of replacement heifers (pure Holstein and F1 crossbred) from birth to first calving are included in the model using genotype-specific biological variables.

To estimate the needed variables for constructing the profit function, a bio-economic simulation model was implemented using MATLAB (version R2025a). The model was carefully designed to avoid double-counting of variables. Revenues and costs are calculated sequentially along the animal’s life cycle and are mutually exclusive: a female calf contributes either as a replacement heifer or as a surplus heifer sold for slaughter, but never both. Feed intake is estimated only once per physiological stage from the NRC [23] energy requirements. Health costs are applied per recorded disease incidence and do not overlap with milk production losses, which are already reflected in reduced milk revenue. Trait interrelationships (e.g., higher birth weight → faster growth → earlier age at first calving; longer calving interval → fewer calves per year → lower replacement rate) are fully incorporated through linked biological sub-models, so that changing one parameter automatically propagates realistic effects to all downstream variables.

The simulation covers the entire productive life of the cow from birth to culling. All revenues, costs, and profit values are expressed on an annual per-cow basis using a steady-state herd structure derived from recorded productive lifetime (PLT) and stage-specific survival rates.

The model integrates biological and economic components of a dairy herd and allows the estimation of profitability for different genotypes. The model structure includes interrelated subsystems representing production performance, reproductive processes, replacement heifer management, herd health, male calf production and sales, and overall management and operational costs. The biological and economic variables incorporated into the model were directly derived from the herd records (Table 1 and Table 2). All data were systematically verified to ensure accuracy regarding growth, reproduction, health, survival rates, and production traits. The average age of the different groups at the start of the model was 57.97 months for Montbéliarde × Holstein, 51.27 months for pure Holstein, and 73.60 months for Simmental × Holstein. These values were extracted from the herd registration data and carefully checked to ensure consistency and reliability.

The simulation model was designed as an integrated system comprising several interrelated subsystems, including: (i) production performance, (ii) revenues and costs associated with milk yield and feed intake, (iii) reproductive processes, (iv) replacement heifer management, (v) herd health and disease control, (vi) male calf production and sales, and (vii) additional managerial and operational aspects.

Along with economic variables, the model incorporated biological indicators, including health status (metabolic disorders, mastitis, digestive diseases, and reproductive disorders), fertility traits (calving interval and age at first calving), productive lifespan, and survival rates across growth stages. These traits were integrated alongside economic measures to provide a holistic bio-economic assessment. In addition to milk sales, revenues from culling and male calves were considered, while expenses encompassed healthcare, veterinary treatments, labor, reproduction, and general management.

The survival rates of calves before and after weaning were derived from actual herd data. The pre-weaning survival rate (SR) was calculated as the number of calves alive at weaning divided by the number of births, and the post-weaning survival rate (PSR) was calculated as the number of calves alive at 365 days of age after weaning divided by the number of calves alive at weaning. These values reflect the actual management conditions at Moghan Agro-Industry and Animal Husbandry Company.

The animal life cycle was divided into five distinct stages: (i) birth to weaning, (ii) weaning to the onset of the fattening phase (12 months), (iii) weaning to the initiation of reproductive activity in heifers (18 months), (iv) first insemination to first calving (replacement heifers), and (v) the productive phase of mature cows over two years of age. Income and cost estimations were conducted over an 18-month calf-rearing period and a 12-month lactation cycle for adult cows.

Nutritional requirements were calculated using equations proposed by the NRC [23], incorporating basal metabolic needs. Feed rations consisted of different combinations of forages, concentrates, forage–concentrate mixtures, silage-based diets, bran, cereal grains, soybean meal, alfalfa, and other protein and energy sources (Table 3).

The model also integrated herd records on calving interval, milk yield, metabolic disorders, mastitis, reproductive disorders, enteritis, digestive diseases, and calf (male and female) performance. Based on the revenues and costs recorded across these components, the economic values of different traits were estimated separately for each herd. The cost structure encompassed feed, veterinary care, labor, health, and management. Net economic values of traits were ultimately determined by subtracting the associated costs from revenues, with results presented separately across age groups.

To estimate the labor, reproductive, and health costs per cow, the standard Cost Accounting method was employed. In this approach, the cost of each item was calculated by multiplying the unit price by the amount consumed or the number of services used per animal. The labor cost per cow was calculated by dividing the total annual labor expenses by the number of cows. The reproductive costs were calculated using the number of annual inseminations and pregnancy diagnoses. Health-related costs were determined according to the expenses for vaccines, antiparasitic drugs, the per-cow share of veterinary visits, and disinfectant materials.

Throughout this manuscript, the term genotype is used exclusively to denote the breed groups under comparison, namely Holstein, Montbéliarde × Holstein, and Simmental × Holstein. No molecular genetic analyses were performed; instead, differences among these breed groups were evaluated based on recorded phenotypic and economic data.

### 2.1. Economic Estimation

#### 2.1.1. Production

Milk production (*MY*) was quantified using individual daily milk yield (kg/day), fat percentage, and lactation length. Annual milk yield for each cow was computed as:

$$dmilk\sum_{d=1}^{305}=MY$$

Fat yield (*FY*) was incorporated using:

FY=MY×fat%

Annual milk revenue was then obtained using:

Rmilk=(MY×Pm)+(FY×Pf)

where *P_m_* is the base milk price ($/kg), and *P_f_* is the price per kg fat.

#### 2.1.2. Feed Requirements and Feed Cost

Daily nutrient requirements were estimated according to the NRC [23]. The equations included maintenance, pregnancy, growth, and lactation energy demands.

$$ER=0.386\times {LW}^{0.75}+(FCM\times 3.14)$$

where *LW* is live weight (kg),$\;{LW}^{0.75}$ is metabolic body weight, and 3.14 MJ/kg *FCM* is the energy cost of milk production.

*DMI* was solved from energy supply equations, and the total daily feed cost was computed as:

Cfeed=(DMIsilage×Psil)+(DMIconc×Pconc)+(DMIforage×Pforage)

where all prices are expressed as $/kg DM.

#### 2.1.3. Health

Health costs included mastitis, metabolic disorders, and lameness. Total health cost was:

$$Chealth=\sum \;{Incidence}_{i}\times \;{TreatmentCost}_{i}+{MilkLoss}_{i}$$

where *MilkLoss_i_* is total milk loss (kg) per case, and Pm is milk price.

The incidence rates of the main health disorders were used as direct input parameters in the bio-economic model. They were extracted directly from the herd health and veterinary records of the studied farm. The incidence of mastitis was recorded as 7%, 5%, and 4% for pure Holstein, Montbéliarde × Holstein, and Simmental × Holstein cows, respectively. The incidence of metabolic disorders (including ketosis and displaced abomasum) was 5%, 4%, and 3% for the same groups, respectively. Lameness incidence was calculated to be 10% in pure Holstein cows, 9% in Montbéliarde × Holstein cows, and 8% in Simmental × Holstein cows. These parameters were incorporated into the model to estimate treatment costs and disease-related milk production losses.

#### 2.1.4. Replacement and Longevity

Productive lifespan (*PLT*), survival rates, and reason for culling were used to determine the annual replacement requirement. Because *PLT* was recorded in days, the replacement rate was computed as:

$$Replacement\;Rate=\frac{1}{PLT}$$

Income from culled cows:

RCull=LWcull×PLW

where *LW_cull_* is live weight at culling (kg), and *PLW* is price per kg live weight. Marketing and transport costs were subtracted when applicable.

#### 2.1.5. Profit Calculation Method

In this study, profit is calculated as the difference between revenues and costs. A complete list of abbreviations used in this section is provided in Table 4. The revenue and cost for each cow per year are calculated as follows [24].

P=R−C

##### Revenue Calculation

The producers’ revenue comes from the sales of milk (*R_milk_*), male calves (*R_male calves_*), culled cows (*R_cows−age_*), and surplus heifers (*R_culled heifers_*), which are calculated according to the following equation:

$$R=R_{malecalves}+R_{culledheifers}+R_{cows-age}+R_{milk}$$

For simplicity, two variables are introduced:

$$NCY=\frac{365}{CI}\;\;and\;PLTY=\frac{PLT}{365}$$

where *NCY* is the number of calvings per cow per year, *CI* is the calving interval (days), *PLTY* is the productive lifetime (years), and *PLT* is the productive lifespan (days).

##### Revenue from Male Calf Sales

Assuming a sex ratio of 0.5, the number of male calves per cow per year is:

NmcCy=0.5×NCY×cr×S24

where *cr* is the calving rate (%), and *S24* is the 24 h survival rate after birth (%). Therefore:

$$R_{male\;calves}=NmcCy\times p_{c}$$

where *p_c_* is the price of a male calf ($ per head).

##### Revenue from Surplus Heifer Sales

A distinction must be made between the proportion of heifers retained in the herd for replacement and the proportion of surplus heifers sold for slaughter. Assuming all female calves are raised and a sex ratio of 0.5, the number of female calves raised per cow per year is:

NfcrCy=0.5×NCY×cr×s24×SR×PSR

where *SR* is the survival rate before weaning (%), and *PSR* is the survival rate after weaning (%).

The number of female calves retained in the herd for replacement per cow per year can be calculated as *1/PLTY*. Therefore, the number of female calves available per cow per year for culling is:

$${NfcCy}_{cull}=NfcrCy-\left(\frac{1}{PLTy}\right)$$

Heifers are sold by weight, so we need the heifer weight, calculated as:

$$W_{heifer}=bw+\left(DG\times wa\right)+(PDG\times dwm)$$

where *bw* is the birth weight of the heifer (kg), *DG* is the daily weight gain before weaning (kg/day), *wa* is the number of days from birth to weaning, *PDG* is the daily weight gain after weaning (kg/day), and *dwm* is the number of days from weaning to 18 months.

Finally,

$$R_{culled\;heifers}={NfcCy}_{cull}\times W_{heifer}\times P_{LW}$$

where *P_LW_* is the price per kg of live weight of culled cows ($).

##### Revenue from Culled Cows

$$R_{cows-age}=\frac{LW}{PLTy}\times P_{LW}$$

where *LW* is the live weight of the culled cow, and *P_LW_* is the price per kg of live weight of culled cows ($).

##### Revenue from Milk Sales

$$R_{milk}=\left(MY\times P_{m}\right)+\left(FY-\left(MY\times 0.035\right)\right)\times P_{f}$$

where *MY* is the amount of milk produced (kg), *p_m_* is the price per kilogram of milk by fat percentage of each group, *FY* is the fat content, and *P_f_* is the price per kilogram of fat.

#### 2.1.6. Variable Cost Calculation

To estimate the feeding costs, animal energy requirements based on live weight were assessed. Considering forage limitations, the model was adjusted as energy requirement changes were reflected in concentrate consumption. Costs are expressed by the following formula:

$$\begin{array}{cc} C= & (C_{Mmalecalves}+C_{Fheifers}+C_{Hheifers}+C_{Rheifers}+C_{Lheifers}+C_{Mculledheifers}+C_{Fcows}+\\ & C_{Hcows}+C_{Rcows}+C_{Lcows}+C_{Mmilk}+C_{Mcows-age})-fixedcost \end{array}$$

where the subscripts refer to products or activities influencing income or cost, and the letters indicate: Feeding (F), Reproduction (R), Health (H), Labor (L), and Marketing activities (M).

##### Marketing Costs of Male Calves (*CM_male calves_*)

$${CM}_{male\;calves}=NmcCy\times M_{calves}$$

where *CM_male calves_* is the marketing cost per male calf.

##### Feeding Cost of Female Calves Until Weaning (*C_Fheifer calves_*)

The number of female calves born and alive within 24 h per cow per year is equal to the number of male calves:

NfcCy=NmcCy

Assuming mortality occurs in the middle of the pre-weaning period:

$$\begin{array}{cc} C_{Fheifer\;calves}= & NfcCy\times \frac{1+SR}{2}\left(\left(56\times 4.5\right)+\left(wa-56\right)\times 3\right)\times p_{m}\\ & +NfcCy\times \frac{1+SR}{2}\times (dm126\times p_{forage}+109.39\times p_{conc}) \end{array}$$

where 56 is the number of days after birth when the amount of milk fed to calves changes from 1.5 kg three times per day to 1.5 kg twice per day; 109.39 kg is the fixed amount of dry matter concentrate consumed by the calves during this period. Here, *p_m_*, *p_forage_*, and *p_conc_* ($) are assumed prices for milk, dry forage, and concentrate, respectively, and *dm126* is the total dry matter forage consumed by calves until weaning (kg). To estimate *dm126*, it was assumed calves start actively consuming forage at 61 days after birth, consuming 0.5% of their body weight in dry matter daily thereafter:

$$dm126=0.005\sum_{i=1}^{65}\left(lw61+\left(i\times DG\right)\right)$$

where

$$lw61=bw+\left(61\times DG\right)$$

##### Feeding Cost of Heifers from Weaning to 18 Months (*C_Fheifer−weaning−18 months_*)

Using average values, the period from weaning to 18 months for a heifer with a growth rate of 0.506 kg/day lasts 414 days. Thus, the feeding cost of heifers from weaning to 18 months is calculated as:

$$\begin{array}{cc} C_{Fheifer-weaning-18\;months} & \\ & =NfcCy\times SR\times \frac{1+PSR}{2}\times (0.33\times dm18)\\ & \times \;p_{sil+\;0.67\times dm18\times p_{forage}+0.024\times p_{conc}\times dwm} \end{array}$$

where *p_sil_* is the price per kg of silage dry matter ($), 0.024 is the fixed daily dry matter concentrate intake (kg), and *dm18* is the total dry matter forage consumed during this period (kg). For *dm18*, it was assumed heifers consume dry matter forage equal to 2% of their body weight, changing daily:

$$dm18=0.02\sum_{i=1}^{540-wa}\left(\left(bw\times DG\right)\right)+(i\times PDG))$$

##### Feeding Cost of Heifers from 18 Months to First Calving Age (*AFC* ($C_{Fheifer-18months-afc})$

This period can be divided into two parts: the first 206 days (*AFC−810*) when heifers are non-pregnant, and the remaining 270 days when pregnant. During the first period, forage dry matter intake is 3% of body weight; during pregnancy, it is 3.5%. With a constant growth rate, total dry matter intake during this period is:

$$dmafc=0.03\sum_{i=1}^{AFC-810}\left(lwafc1\right)+0.035\sum_{i=1}^{270}\left(lwafc2\right)$$

where *lwafc1* and *lwafc2* are calculated as:

$$lwafc1=W_{heifer}+\left[i\times \left(\frac{\left(LW-W_{heifer}\right)}{\left(AFC-540\right)}\right)\right]$$

$$lwafc2=\left\{W_{heifer}+\left[\left(AFC-810\right)\times \left(\frac{\left(LW-W_{heifer}\right)}{\left(AFC-540\right)}\right)\right]\right\}+\left[i\times \left(\frac{\left(LW-W_{heifer}\right)}{\left(AFC-540\right)}\right)\right]$$

The feeding cost (forage and concentrate) of heifers from 18 months to *AFC* is:

$$C_{Fheifer-18\;months-afc}=\frac{1}{PLTy}\times (0.33\times dmafc\times p_{sil}+0.67\times dmafc\times p_{forage}\;+0.89\times p_{conc}\times dafc)$$

where *dafc = AFC − 540* days.

##### Total Feeding Costs of Heifers from Birth to First Calving (*C_Fheifer_*)


$$C_{Fheifer}=C_{Fheifer\;calves}+C_{Fheifer-weaning-18\;months}+C_{Fheifer-18\;months-afc}$$

##### Health Costs of Heifers from Birth to Weaning (*C_Hheifer_* _*calves*_)

$$C_{Hheifer\;calves}=NfcCy\times \frac{1+SR}{2}\times wa\times c_{Hhealth}$$

where *C_Hhealth_* is the daily health cost per heifer ($).

##### Health Costs of Heifers from Weaning to 18 Months ($C_{Hheifer-weaning-18months}$)


$$C_{Hheifer-weaning-18\;months}=NfcCy\times SR\times \frac{1+PRS}{2}\times dwm\times c_{Hhealth}$$

##### Health Costs of Heifers from 18 Months to First Calving ($C_{Hheifer-18months-afc}$)


$$C_{Hheifer-18\;months-afc}=\frac{1}{PLTy}\times dafc\times c_{Hhealth}$$

##### Total Health Costs of Heifers from Birth to First Calving (*CHheifer*)


$$C_{Hheifer}=C_{Hheifer\;calves}+C_{Hheifer-weaning-18\;months}+C_{Hheifer-18\;months-afc}$$

##### Labor Costs of Heifers (*C_Lheifers_*)

The labor costs of heifers (*C_Lheifers_*) from birth until the first calving were calculated similarly to the method used for heifers, with the difference that in the above equations, the health costs of heifers (*C_Hhealth_*) were replaced by the daily labor cost per heifer (clabour).

##### Reproductive Costs of Heifers (*C_Rheifers_*)

$$C_{Rheifers}=\frac{1}{PLTy}\times dafc\times c_{Hrepro}$$

where *C_Hrepro_* is the daily reproductive cost of the heifer ($).

##### Marketing Costs of Heifers ($C_{Mculled\;heifers}$)

$$C_{Mculled\;heifers}={NfcCy}_{cull}\times W_{heifer}\times m_{LW}$$

where *m_LW_* is the marketing cost per kilogram of mature live weight (in $).

##### Feeding Costs per Cow (*C_Fcows_*)

$$C_{Fcows}=365\times FCD$$

where *FCD* is the daily feed cost calculated as:

$$FCD=conc\times p_{conc}+0.33\times sil\times p_{sil}+0.67\times {for}_{milk}\times p_{forage}$$

where *conc* is the amount of dry matter concentrate consumed per day (2.35 kg); *sil* is the amount of dry matter from dry forage consumed per day during the dry period (15 kg dry matter/day); and *for_milk_* is the amount of dry matter forage consumed per day (kg). *Foragemilk* was calculated from *MY*, *FY*, and *LW*, assuming the cows were in energy balance.

The energy requirement (*ER*, in MJ of net lactation energy per day) for maintenance and milk production was estimated using the following equation:

$$ER=\left({0.293LW}^{0.75}+3.05FCM\right)\times \left(0.9752+0.00165FCM\right)$$

where *LW* is the mature live weight; *FCM* is fat-corrected milk (kg/day) calculated according to NRC [23] as:

FCM=(1/365)(0.4MY+15FY)

The energy intake from forages specifically consumed to support milk production (hereafter referred to as *foragemilk*) was estimated as part of daily dry matter intake. Thus, the total daily energy intake from forages (*EIP*) was calculated as:

EIP=ER−EIC

where *EIC* is the energy intake from concentrates ($7.19=\;MJ\;{NE}_{L}$).

The dry matter intake and energy capacity of forages, *foragemilk* (kg dry matter), was estimated as:

EIP5.65

##### Milk Marketing Costs (*C_Mmilk_*)

$$C_{Mmilk}=MY\times M_{milk}$$

where *M_milk_* is the marketing cost per kilogram of milk.

##### Marketing Costs of Culled Cows (*C_Mcows-age_*)


$$C_{Mcows-age}=\frac{LW}{PLTy}\times m_{LW}$$

The economic coefficient for each trait (milk yield, calving interval, birth weight, pre-weaning gain, and post-weaning gain) was calculated as the difference in profit between the modified state and the baseline. This procedure enabled quantification of the marginal economic contribution of each trait to overall herd profitability. The economic coefficient of a given trait was estimated using the following general equation:

$$V_{i}=(p_{\mu i+∆}-p_{\mu i})/∆$$

$V_{i}$ is the economic coefficient,

$p_{\mu i+∆}$ is the average profit per animal after a one-unit increase in trait i,

$p_{\mu i}$ is the average profit per animal before the change in the mean,

∆ is the amount of increase in the mean of trait i.

**Figure 1:**
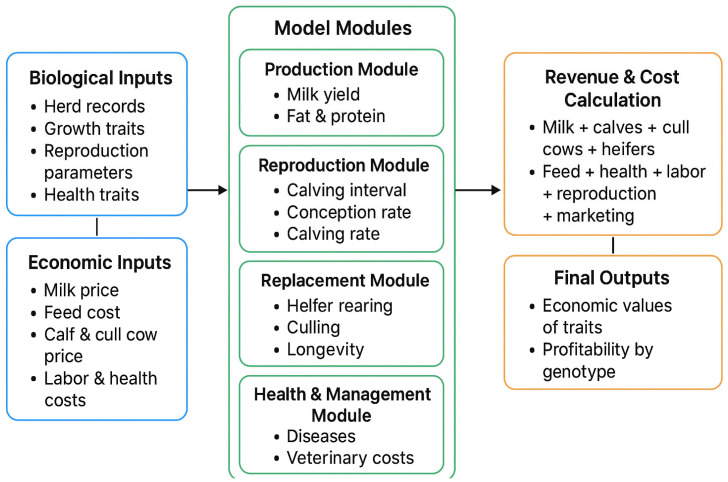
Flowchart of the bio-economic simulation model applied in this study.

In this study, we used MATLAB (version R2025a) to create a simulation model of the bio-economic system of a dairy herd. By using this model, we tried to determine both the costs and the income from managing the herd. Annual profits were computed for each age group (A to P), and a weighted average profit was calculated according to the relative contribution of each age group to the herd structure. A marginal approach was used to determine the economic coefficients of each trait. In particular, the mean value of the trait of interest was raised by one unit while considering all other traits at their population means. Figure 1 shows the flowchart of the bio-economic simulation model for estimating economic coefficients of dairy cattle traits.

## 3. Results

### 3.1. System Revenues Evaluation

Based on the results presented in Table 5, differences in revenue components were observed among the three genotypes. Milk sales revenue was higher in the Montbéliarde × Holstein crossbred group ($2223) compared with pure Holstein ($2123) and the Simmental × Holstein crossbred group ($1810). These results indicate that the genotypes differ in milk production, with the Simmental × Holstein group showing relatively lower revenue for this trait.

Regarding male calf sales, both crossbred groups outperformed pure Holsteins. As shown in Table 5, revenues were nearly identical in Montbéliarde × Holstein and Simmental × Holstein crosses ($241 and $240, respectively), whereas pure Holstein yielded a considerably lower figure ($195). This result demonstrates that crossbreeding, particularly with Montbéliarde and Simmental, enhances the economic value of male calves and thus contributes to improving revenue diversification.

In contrast, revenues from surplus heifer sales were highest in pure Holstein herds ($159). Montbéliarde × Holstein crossbreds recorded the lowest value ($103), suggesting that the introduction of Montbéliarde genetics reduces either the number or the market value of surplus heifers relative to pure Holstein.

**Figure 2:**
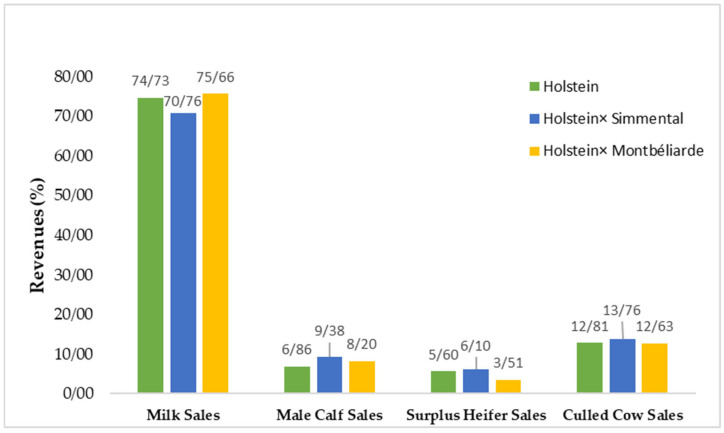
Annual revenues from milk and animal sales across purebred and crossbred genotypes.

Regarding culled cow sales, differences among genotypes were relatively minor. Nevertheless, as depicted in Figure 2, Montbéliarde × Holstein exhibited a slight advantage ($371), whereas Simmental × Holstein reported the lowest revenue ($352).

### 3.2. System Cost Evaluation

**Figure 3:**
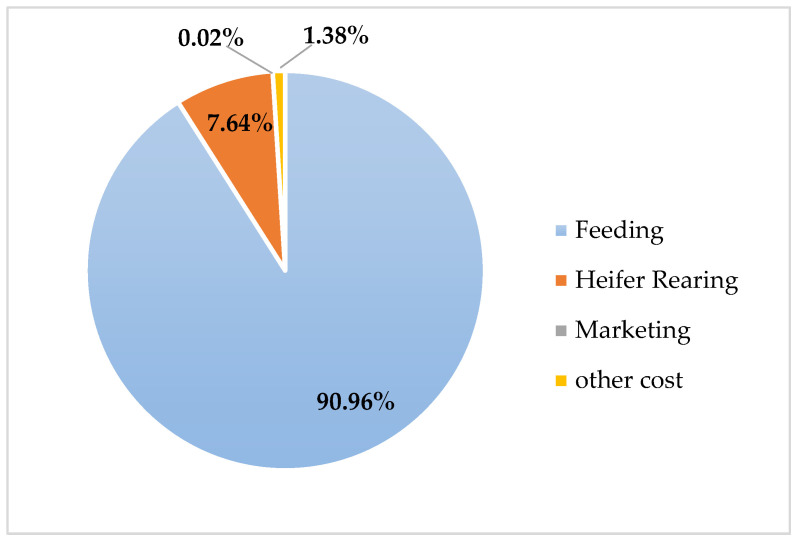
Percentage distribution of annual costs across the Holstein genotype.

**Figure 4:**
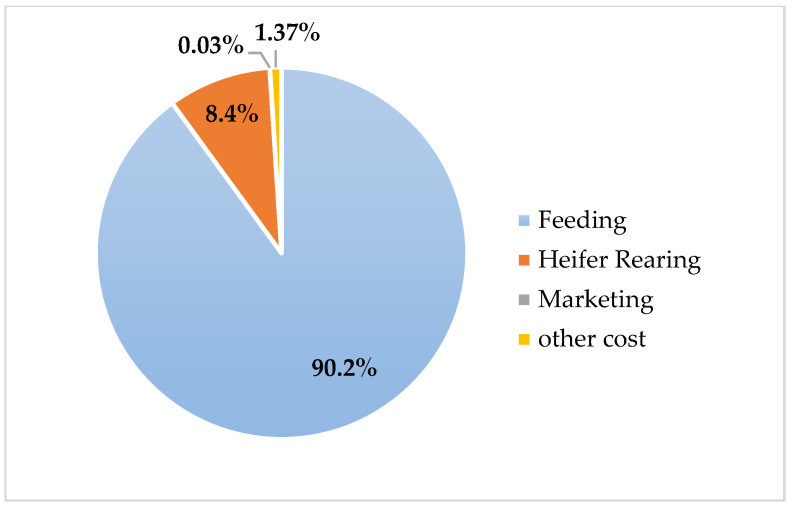
Percentage distribution of annual costs across the Simmental × Holstein genotype.

**Figure 5:**
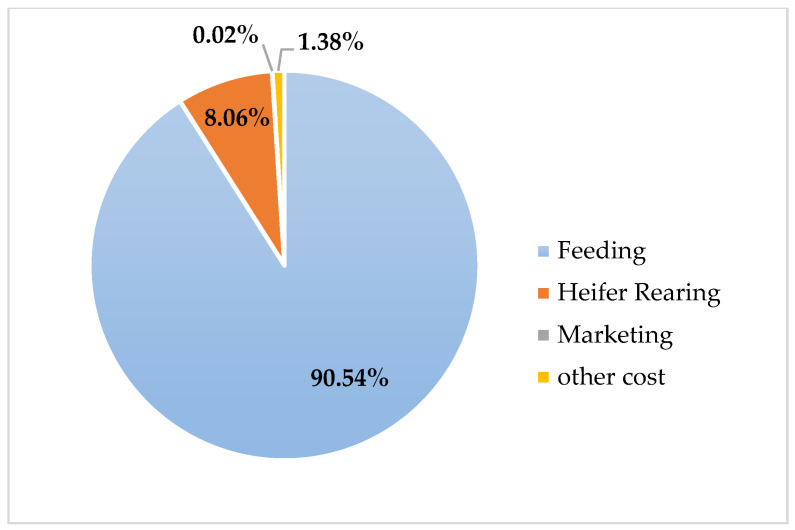
Percentage distribution of annual costs across the Montbéliarde × Holstein genotype.

The comparative evaluation of annual maintenance costs among pure Holstein, Simmental × Holstein, and Montbéliarde × Holstein genotypes revealed significant economic differences (Table 6). Feed costs constituted the most significant component of total expenses. They were highest in pure Holstein herds, amounting to $1774. In contrast, the crossbred groups had lower feeding costs, with Simmental × Holstein showing the lowest ($1470) and Montbéliarde × Holstein showing an intermediate value ($1585). A similar pattern was observed for heifer rearing, where pure Holstein incurred the most significant expense ($149), followed by Montbéliarde crosses, while Simmental crosses had the lowest rearing costs ($137). The percentage distribution of cost categories for each genotype is visualized in Figure 3, Figure 4 and Figure 5, clearly demonstrating the predominance of feeding costs across all groups.

Marketing expenses showed negligible variation among the three genetic groups, indicating no significant impact of breed type on this cost category. However, other costs, including miscellaneous management and healthcare expenditures, were again highest in pure Holstein ($26.88) and lowest in Simmental × Holstein ($22.47).

Total variable costs further underscored the economic advantage of crossbreeding. Pure Holstein herds incurred the highest costs ($2139), whereas Simmental × Holstein ($1663) and Montbéliarde × Holstein ($1751) achieved reductions of approximately 22% and 18%, respectively, relative to pure Holstein. Fixed costs were constant across all genotypes ($187). The proportional contribution of feeding to total variable costs is illustrated in Figure 3 (Holstein), Figure 4 (Simmental × Holstein), and Figure 5 (Montbéliarde × Holstein).

### 3.3. Annual Profitability

The results demonstrated that annual profitability was positive across all three genetic groups, confirming the economic viability of each production system (Table 7). Pure Holstein herds exhibited the highest total costs ($2138) alongside total revenues of $2699, yielding a net profit of $561. In comparison, the Simmental × Holstein crossbred group incurred lower total costs ($1817) and generated revenues of $2560, resulting in a net profit of $743. Although the Montbéliarde × Holstein crossbred group incurred higher production costs ($1938), its high total revenue ($2940) resulted in the greatest net profit ($1002) among all groups.

**Figure 6:**
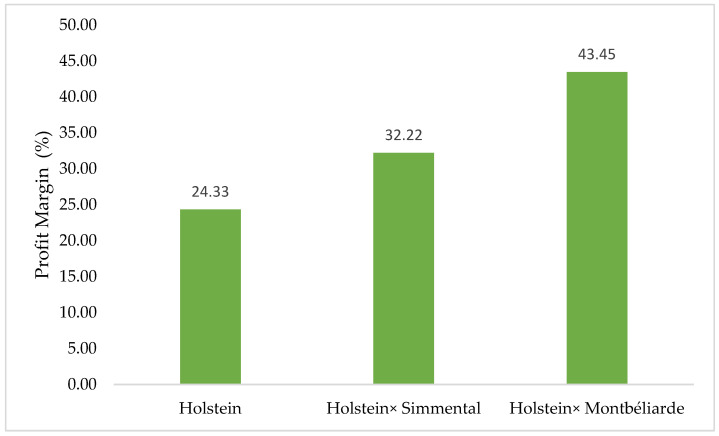
Percentage distribution of annual profit margin in the studied genotypes.

As illustrated in Figure 6, the percentage distribution of annual profit margin differed among the genotypes, with Montbéliarde × Holstein achieving the largest economic advantage, followed by Simmental × Holstein and pure Holstein. These findings clearly highlight that crossbreeding provides superior economic returns compared with pure Holstein herds. The enhanced profitability of Simmental × Holstein crossbreds is mainly driven by reduced production costs, while Montbéliarde × Holstein crossbreds benefit from greater revenue generation. Generally, the Montbéliarde crossbreeding system demonstrated better profitability, emphasizing the value of targeted crossbreeding strategies for optimizing economic performance in the dairy cattle industry.

### 3.4. Economic Value of Traits

The economic evaluation of traits across pure Holstein, Montbéliarde × Holstein, and Simmental × Holstein genotypes revealed distinct patterns, underscoring the significance of crossbreeding strategies in optimizing the dairy production systems (Table 8). For milk yield, the Montbéliarde × Holstein crossbred exhibited the highest absolute economic value ($0.064) and economic weight ($41.46), outperforming both Holstein ($0.049; $30.19) and Simmental × Holstein ($0.040; $36.00). These results indicate that the incorporation of Montbéliarde genetics substantially enhances the profitability of milk production. This finding is consistent with previous studies reporting that Montbéliarde crosses improve milk solids and overall economic returns compared with pure Holstein herds.

In contrast, the calving interval displayed consistently negative economic values across all genotypes, reflecting its unfavorable impact on profitability. While Holstein (−$0.77; −$10.06) and Montbéliarde × Holstein (−$0.98; −$11.34) were associated with relatively moderate economic losses, the Simmental × Holstein cross incurred a markedly greater economic penalty (−$0.94; −$148.20). These results highlight the heightened sensitivity of Simmental crosses to reproductive inefficiencies, reinforcing the importance of rigorous reproductive management in such herds.

For birth weight, both crossbred genotypes demonstrated superior economic performance compared with pure Holstein. The economic value for Holstein was minimal ($0.03; $0.06), whereas Montbéliarde × Holstein ($0.23; $0.40) and Simmental × Holstein ($0.39; $0.87) yielded substantially higher returns. This improvement can be attributed to the genetic contribution of dual-purpose breeds, which are characterized by enhanced growth potential and greater calf viability.

A comparable pattern was observed in pre-weaning daily gain, where pure Holsteins exhibited the lowest economic values ($0.002 and $0.06). In contrast, Montbéliarde × Holstein ($0.017; $0.41) and Simmental × Holstein ($0.025; $0.64) showed significant economic advantages. These findings underscore the role of crossbreeding in enhancing early growth performance, thereby increasing calf-rearing efficiency and reducing costs linked to prolonged rearing periods.

The superiority of the Simmental cross was particularly evident in post-weaning daily gain, where its economic weight ($3.96) far exceeded that of Montbéliarde × Holstein ($2.47) and pure Holstein ($0.44). This outcome reflects the strong growth potential of Simmental genetics, which contributes to improved market weights and overall profitability.

## 4. Discussion

The present study indicated that purebred Holstein cows ranked third in economic profitability, whereas Montbéliarde × Holstein crossbred cows achieved the highest net profit among the examined genotypes. These results are consistent with previous findings and highlight the importance of crossbreeding strategies in enhancing herd economic performance [25,26,27]. Several studies have shown that crossbred cows, particularly dual- or triple-cross combinations, exhibit superior economic and productive performance compared to purebred Holsteins. For instance, Hazel et al. (2021) reported that Holstein × Viking Red and Montbéliarde × Holstein crossbreds in Minnesota had 13% and 9% higher daily profits, respectively, than purebred Holsteins, primarily due to improved fertility, lifetime production, and animal health [19]. Similarly, Dezetter et al. (2017) demonstrated that introducing crossbred cows into herds, even when comprising only 30% of the herd, generated significant economic returns [8]. Field studies in Sweden, New Zealand, and Italy further confirm that rotational crossbreeding with Viking Red, Montbéliarde, and Jersey sires improves net income over feed costs, reduces culling risk, and enhances cheese yield potential in crossbred herds compared to pure Holsteins [28,29]. These results align with studies by Knob et al. (2023) that showed Montbéliarde × Holstein crossbreds outperform purebred Holsteins in fertility, longevity, and milk quality, even if milk yield is slightly lower, ultimately resulting in higher profitability [30]. Simulation studies in France also showed that crossbreeding Holsteins with Swedish Red, Montbéliarde, and Normandy breeds substantially increased profitability, mainly through enhanced fertility, health, and production performance [18].

This superior economic performance of Montbéliarde × Holstein and other crossbreds is consistently attributed to heterosis effects on functional traits, even when milk volume is slightly lower [29,31]. Crossbreds, including Swedish Red × Holstein [28] and Montbéliarde-sired progeny [32], exhibit higher conception rates, fewer services per conception, shorter calving intervals, lower mortality and involuntary culling rates, younger age at first calving, and higher lifetime productivity. Additionally, crossbred cows typically achieve greater cull cow value at the end of their productive life [33] and, when combined with genomic testing and sexed semen, deliver substantial overall economic returns [18,34]. Although purebred Holsteins often excel in milk volume under intensive systems, crossbreds frequently compensate with superior milk solids and technological properties. Rotational three-breed crosses and F1 Montbéliarde × Holstein cows commonly produce milk with higher protein, casein, and fat content, as well as better coagulation properties and cheese-making quality [31]. Particular crosses, such as Simmental × Holstein F1, also show improved energy-corrected milk yield and better metabolic adaptation during the transition period [26]. However, environmental and climatic factors can modulate crossbreeding outcomes. In hot and humid conditions, Montbéliarde × Holstein and Simmental × Holstein crosses do not consistently outperform purebred Holsteins and may exhibit increased calving difficulty [35]. In subtropical regions (e.g., Egypt and Brazil), Fleckvieh, Brown Swiss, or other dual-purpose breeds display lower culling rates and superior reproductive performance under heat stress [36,37,38].

We observed negative values for calving interval across all genotypes (−$14.20, −$11.34, and −$10.06 for Simmental × Holstein, Montbéliarde × Holstein, and pure Holstein, respectively), indicating that annual profit per cow decreases as the interval between successive calvings lengthens. This reduction primarily stems from lower annual milk, fat, and protein yields, fewer lifetime calvings, and reduced revenue from surplus animals, which outweigh the modest savings in feed and veterinary costs. These results are in strong agreement with the majority of published studies that report negative economic weights for calving interval [39,40,41]. For example, Sadeghi-Sefidmazgi et al. (2012) estimated an economic loss of approximately −$0.72 per additional day of calving interval in Iranian Holsteins [39], while bio-economic models in Germany and China derived similarly negative weights [13,22]. Simulation studies further confirm that a target calving interval close to 12 months maximizes herd profitability and sustainability [42]. Although a few studies have reported marginally positive values [24], these exceptions are generally attributable to methodological limitations, particularly the omission of reduced annual milk yield in the profit function.

All genotypes exhibited positive economic values for birth weight, with the highest contribution observed in Simmental × Holstein crossbreds due to substantially greater market value of male calves and surplus heifers sold for meat or breeding purposes. Although the absolute values estimated here were higher than those previously reported by Shadparvar et al. [43], the positive direction is entirely consistent with studies that incorporate calf revenue into profit equations. Nevertheless, the literature consistently highlights a critical trade-off: while moderate increases in birth weight enhance calf value and may even be associated with higher subsequent milk production within an optimal range [44], excessive birth weight dramatically elevates the risk of dystocia and its associated economic losses [45,46]. Each additional kilogram of birth weight has been shown to increase the odds of dystocia by approximately 13% in Holsteins [47], leading to reduced milk solids yield, prolonged days open, higher services per conception, increased cow mortality, and elevated veterinary costs [46]. Average birth weights reported for Holstein calves typically range from 37.1 to 41.4 kg, with male and multiparous-born calves being heavier [48,49].

Positive economic values were observed for daily weight gain across all genotypes, both pre- and post-weaning. Profitability was highest for Simmental × Holstein ($0.64 before weaning and $3.96 after weaning), followed by Montbéliarde × Holstein ($0.41 and $2.47), and lowest for pure Holsteins ($0.06 and $0.44). These results underscore the importance of selecting for improved growth performance to maximize production efficiency, particularly in crossbred populations. Although some discrepancies exist, such as Athari-Mortazavi et al. (2010), who reported negative values due to differing bio-economic modeling assumptions [50], these findings are generally in agreement with those of Kahi and Nitter [24] and Sahragard et al. [41]. Studies on organic and grass-based systems further demonstrate that crossbred calves fed whole milk or under ad libitum regimes exhibit superior growth rates and economic returns during rearing [24,41], aligning with the observed advantages in pre- and post-weaning gains [51]. The present study’s findings regarding the positive economic values for daily weight gain across all genotypes are consistent with the existing scientific literature. Research consistently shows that crossbred calves exhibit superior growth performance compared to purebreds. For instance, studies comparing Horro (Zebu) cattle with their Holstein–Friesian–Horro and Jersey–Horro crosses found higher growth performance in crossbred calves [52]. Similarly, Belgian Blue crossbreds have been shown to outperform Kedah-Kelantan and Brahman breeds by 50–100% in live weight gains under tropical conditions, demonstrating the advantages of heterosis for upgrading local herds [53]. In another study, crossbred calves (Holstein, Montbéliarde, and Swedish Red combinations) showed similar or superior growth rates compared to pure Holsteins, especially when fed ad libitum milk allowances [54]. The economic benefits of improved growth are evident, as higher daily gains can lead to earlier slaughter ages. The superior profitability observed for Simmental × Holstein and Montbéliarde × Holstein crossbreds in daily weight gain is supported by studies indicating that crossbreeding can enhance various performance traits. Simmental crossbred cattle have shown higher growth performance and improved carcass characteristics compared to local breeds [55]. Simmental × Holstein cows have also been found to maintain comparable feed efficiency and milk production to purebred Holsteins, while being more resilient to heat stress, making them suitable for high-production systems [30]. Montbéliarde × Holstein crossbreds have demonstrated higher average milk production and protein content, along with lower somatic cell scores, indicating overall improved efficiency [56]. While pure Holsteins are known for high milk yield, crossbreeding with breeds such as Simmental and Montbéliarde can introduce beneficial traits including improved growth rates, fertility, and health that collectively enhance economic profitability [11,15].

The observation that crossbred calves fed whole milk or under ad libitum regimes exhibit superior growth rates and economic returns is also supported by previous studies. Feeding whole milk, particularly ad libitum, has been shown to result in higher weaning weights and better post-weaning growth [57]. Calves on ad libitum milk feeding demonstrate greater average daily gains compared to those on restricted allowances. Although ad libitum feeding can increase milk costs, the cost per kilogram of gain may be comparable or even more favorable given enhanced growth performance [54]. In organic systems, feeding whole milk to calves is more cost-effective than milk replacers, without compromising growth [58]. While the bio-economic model used in this study provides a practical tool for economic comparison of genotypes, it is essential to acknowledge its limitations. The model relies on observed biological parameters and operational conditions and does not account for long-term price fluctuations, environmental risks, or population dynamics. Bio-economic models are valuable for integrating biological and economic factors to assess production systems and estimate economic weights for traits. However, their accuracy depends on underlying assumptions and the variables included. For instance, periodic recalibration is necessary to reflect current market conditions [59]. Additionally, the models may not fully capture real-world complexities, such as environmental stressors or dynamic markets [60]. Therefore, while these models provide important guidance for herd-level economic decisions, they should be interpreted with an understanding of their inherent limitations and may be complemented by more comprehensive dynamic or cost–benefit analyses [61].

## 5. Conclusions

The results of this study indicate that crossbreeding Holstein cows with Montbéliarde or Simmental breeds can enhance economic performance and milk production under specific production conditions. Simmental × Holstein crossbreds benefited from lower operational costs and higher revenues from male calves, whereas Montbéliarde × Holstein crossbreds exhibited the highest milk revenues. Both types of crossbreds showed improvements in birth weight and growth performance compared to pure Holsteins, with Montbéliarde crosses achieving higher overall profitability in this study. However, the simplicity of the model employed may not fully capture all aspects of herd population dynamics, and the results are largely sensitive to input parameters. Consequently, variations in key biological and managerial parameters can influence the predicted economic outcomes. These limitations should be taken into account when interpreting results and designing crossbreeding strategies. Overall, the findings suggest that targeted crossbreeding can serve as an effective strategy to enhance sustainability and profitability in dairy production systems, provided that breeding strategies are carefully tailored to local management practices, production systems, and market conditions.

## Figures and Tables

**Table 1:** Biological Variables Used for Modeling Based on Data from Moghan Agro-Industry.

Variable	Holstein	Montbéliarde × Holstein	Simmental × Holstein
Birth weight (kg)	35.5 ± 5.17	38.0 ± 4.39	40.0 ± 6.09
Daily weight gain before weaning (g)	700 ± 95.05	750 ± 101.25	750 ± 118.36
Daily weight gain after weaning (g)	750 ± 75.35	850 ± 89.36	950 ± 102.45
Adult body weight (kg)	598 ± 38.63	667 ± 55.36	847 ± 45.80
Body condition score (1 to 5)	3.0 ± 0.14	3.75 ± 0.20	4.25 ± 0.19
Calving interval (days)	440 ± 56.73	394 ± 36.95	407 ± 45.25
Age at first calving (days)	769 ± 45.75	768 ± 42.58	757 ± 39.68
Productive lifespan (days)	1058 ± 368.78	1310 ± 478.15	1752 ± 631.45
Calving rate (%)	95 ± 0.36	95 ± 0.18	97 ± 0.85
Conception rate (%)	47.32 ± 0.97	48.87 ± 1.23	53.32 ± 1.01
Number of services per conception	2.2 ± 0.08	1.8 ± 0.05	1.9 ± 0.09
Calf mortality (0–24 h) (%)	2.26 ± 0.07	1.98 ± 0.08	1.76 ± 0.04
Annual milk yield per cow (kg)	10,506 ± 1243	7520 ± 1300	5320 ± 1800
Annual fat yield per cow (kg)	340.4 ± 18.52	293.4 ± 14.25	247.4 ± 14.74
Milk fat (%)	3.23 ± 0.04	3.90 ± 0.05	4.46 ± 0.02
Milk protein (%)	3.23 ± 0.02	3.60 ± 0.01	3.50 ± 0.02
Survival rate before weaning (%)	95 ± 1.01	93 ± 1.28	95 ± 1.42
Survival rate after weaning (%)	98 ± 0.96	97 ± 0.12	98 ± 0.27
Survival rate 24 h after birth (%)	98 ± 1.19	95 ± 1.07	98 ± 1.08
Daily dry silage consumption (kg)	20.0	19.5	13.0
Daily dry concentrate consumption (kg)	12.5	9.5	9.5

**Table 2:** Economic Variables Used for Modeling Based on Data from Moghan Agro-Industry.

Variable	Holstein	Montbéliarde × Holstein	Simmental × Holstein
Price per kg of milk ($)	0.29	0.29	0.29
Price per kg of fat ($)	6.00	6.00	6.00
Price per kg of dry matter silage ($)	0.08	0.08	0.08
Price per kg of dry matter concentrate ($)	0.33	0.33	0.33
Price per kg of dry matter forage ($)	0.19	0.19	0.19
Price per kg live weight of culled cows ($)	1.88	2.13	2.13
Daily health cost per heifer ($)	0.015	0.015	0.015
Daily labor cost per heifer ($)	0.075	0.075	0.075
Daily reproductive cost per heifer ($)	0.018	0.021	0.021

**Table 3:** Representative daily feed ration and chemical composition of diets (kg DM basis) for three dairy cattle genotypes.

Ingredient (kg DM/day)	Holstein	Montbéliarde × Holstein	Simmental × Holstein
Corn silage	20.00	19.50	13.00
Alfalfa hay	3.00	2.50	2.00
Corn grain	5.10	4.30	4.00
Barley grain	3.40	2.30	2.50
Soybean meal (44% CP)	2.50	1.80	1.80
Wheat bran	0.80	0.60	0.65
Canola meal	0.50	0.35	0.40
Mineral–vitamin mix & salt	0.20	0.15	0.15
**Chemical composition (% of DM)**			
Crude protein (CP)	16.00	16.21	16.51
Neutral detergent fiber (NDF)	34.50	33.80	32.00
Total digestible nutrients (TDN)	71.01	71.31	72.01

**Table 4:** Explanation of abbreviations and technical terms used in the Section 2.

Full Name	Abbreviation
Milk yield (kg/cow/year)	MY
Fat yield (kg/cow/year)	FY
Fat-corrected milk	FCM
Calving interval (days)	CI
Productive lifetime (years)	PLT
Productive lifetime (years)—used in replacement calculations	PLTY
Number of calvings per cow per year	NCY
Pre-weaning survival rate of calves (%)	SR
Post-weaning survival rate of calves (%)	PSR
Survival rate 24 h after birth (%)	S24
Daily weight gain before weaning (kg/day)	DG
Daily weight gain after weaning (kg/day)	PDG
Age at first calving (days)	AFC
Dry matter intake (kg/day)	DMI
National Research Council (Nutrient Requirements of Dairy Cattle)	NRC
Birth weight (kg)	BW
Number of days from birth to weaning	wa
Number of days from weaning to 18 months of age	dwm
Live weight of culled cow (kg)	LW
Price per kg live weight of culled animals ($/kg)	PLW
Price per kg of milk ($/kg)	pm
Price per kg of fat ($/kg)	pf
Price of a male calf ($/head)	pcp
Calving rate (%)	CR
Revenue from milk sales ($/cow/year)	Rmilk
Revenue from male calf sales ($/cow/year)	Rmale calves
Revenue from surplus heifer sales ($/cow/year)	Rculled heifers
Revenue from culled cow sales ($/cow/year)	Rcows-age
Feeding cost of lactating cows ($/cow/year)	CFcows
Total feeding cost of replacement heifers from birth to first calving ($/heifer)	CFheifer
Total health cost of replacement heifers ($/heifer)	CHheifer
Labor cost of replacement heifers ($/heifer)	CLheifers
Reproductive cost of heifers ($/heifer)	CRheifers
Marketing cost of milk ($/cow/year)	CMmilk
Marketing cost of culled cows ($/cow/year)	CMcows-age
Marketing cost of male calves ($/cow/year)	CMmale calves

**Table 5:** Comparison of annual revenue items among Holstein, Simmental × Holstein, and Montbéliarde × Holstein cows.

Variable	Holstein	Simmental × Holstein	Montbéliarde × Holstein
Milk Sales	2123	1810	2223
Male Calf Sales	195	240	241
Surplus Heifer Sales	159	156	103
Culled Cow Sales	364	352	371

**Table 6:** Comparison of annual costs of Holstein, Simmental × Holstein, and Montbéliarde × Holstein cows.

Variables	Holstein	Simmental × Holstein	Montbéliarde × Holstein
Feeding	1774	1470	1585
Heifer Rearing	149	137	141
Marketing	0.40	0.45	0.43
Other Costs	26.88	22.47	24.14
Variable Costs	1951	1630	1751
Fixed Costs	187	187	187

**Table 7:** Annual profitability of Holstein, Simmental × Holstein, and Montbéliarde × Holstein cows ($).

Variables	Holstein	Simmental × Holstein	Montbéliarde × Holstein
Total Costs	2138	1817	1938
Total Revenue	2699	2560	2940
Net Profit	561	743	1002

**Table 8:** Economic Weights with both Absolute Economic Value & Economic Weight in $.

Trait	Absolute Economic Value ($)	Genetic Standard Deviation	Economic Weight ($)
**Milk yield**			
Holstein	0.049	621	30.19
Montbéliarde × Holstein	0.064	650	41.46
Simmental × Holstein	0.040	900	36.00
**Calving interval**			
Holstein	−0.77	16.13	−10.06
Montbéliarde × Holstein	−0.98	11.61	−11.34
Simmental × Holstein	−0.94	15.77	−14.20
**Birth weight**			
Holstein	0.03	1.86	0.06
Montbéliarde × Holstein	0.23	1.70	0.40
Simmental × Holstein	0.39	2.22	0.87
**Pre-weaning gain**			
Holstein	0.002	10.24	0.06
Montbéliarde × Holstein	0.017	23.70	0.41
Simmental × Holstein	0.025	25.02	0.64
**Post-weaning gain**			
Holstein	0.016	27.80	0.44
Montbéliarde × Holstein	0.097	25.60	2.47
Simmental × Holstein	0.141	28.20	3.96

## Data Availability

The data that support the study findings were obtained under license and thus were not deposited in an official repository. Information can be made available from the authors upon request. In the preparation of this article, no artificial intelligence technologies or tools were directly used; only very limited use was made for language editing and improvement.
